# Transgenerational Genetic Effects Help Explain Latitudinal Variation in Seed Mass and Germination Timing in *Plantago lanceolata*

**DOI:** 10.3390/plants11040522

**Published:** 2022-02-15

**Authors:** Elizabeth P. Lacey, Matthew M. Marshall, Marc Bucciarelli, Scott J. Richter

**Affiliations:** 1 Department of Biology, University of North Carolina, P.O. Box 26170, Greensboro, NC 27402, USA; marshall@alumni.uncg.edu (M.M.M.); mdbuccia@gmail.com (M.B.); 2 Bioinformatics Department, J Michael Consulting, LLC, 885 Woodstock Rd., Roswell, GA 30075, USA; 3 Department of Mathematics & Statistics, University of North Carolina, P.O. Box 26170, Greensboro, NC 27402, USA; sjricht2@uncg.edu

**Keywords:** transgenerational effects, maternal genetic effects, latitude, seed, germination, *Plantago lanceolata*, species conservation

## Abstract

We know little about the underlying genetic control of phenotypic patterns of seed traits across large-scale geographic and environmental gradients. Such knowledge is important for understanding the evolution of populations within species and for improving species conservation. Therefore, to test for genetic variation in *Plantago lanceolata*, we made reciprocal crosses between northern and southern genotypes that span the species’ range in Europe. The results provide evidence of transgenerational genetic effects on seed mass and germination timing. Northern mothers produced larger seeds with delayed germination, in contrast to southern mothers, which produced smaller seeds with accelerated germination. A maternal latitude affected both the seed coat, solely maternal tissue, and embryo/endosperm tissues. Thus, latitudinal variation in seed size and germination timing can be explained, in part, by the direct influence of maternal genotype, independent of zygotic genes that parents pass directly to the embryo and endosperm. Data suggest that researchers exploring the existence and evolution of large-scale geographic variation within species test for transgenerational genetic effects. In addition, data suggest that transgenerational control of seed traits should be considered when developing procedures designed to facilitate species conservation and restoration.

## 1. Introduction

Large-scale geographic variation has long been a focus of plant research [1,2,3,4,5], and much of that research has focused on seed traits because of their large contributions to a species’ fitness. Seed mass can impact seed germination, seedling establishment [6,7,8,9], and dispersal [10,11,12,13]. Germination timing, including dormancy, can strongly influence seedling establishment, survivorship, and reproduction, which contribute to lifetime fitness [9,12,14,15,16,17,18,19,20,21]. For all these reasons, seed traits are also key determinants of agronomic success [22].

These contributions to fitness have motivated many evolutionary biologists to explore how seed traits vary systematically along latitudinal/altitudinal/longitudinal gradients. In addition, conservation biologists studying the effects of large-scale environmental change (e.g., via climate change, urbanization, agriculture, and deforestation) have increased efforts to sample and preserve seeds from multiple populations along these gradients. Seed traits can influence a species’ ability to shift its range, recover from disturbance, and adapt to local warming and increased drought in response to climate change. Thus, understanding the underlying causes of large-scale variation (e.g., mass, germination, dormancy, and longevity) is expected to help reduce the probability of species extinction and expand opportunities for crop improvement [23,24,25,26,27,28].

Thus far, large-scale studies have demonstrated that populations across the geographic range of a species can vary phenotypically in seed mass and germination. However, patterns are quite variable. In some species, mass declines poleward [26,29,30,31,32,33,34,35,36,37,38,39,40,41]. In others, mass increases poleward [33,40,42,43,44,45,46,47,48,49], and with increasing altitude [30,50,51,52,53] or shows no geographic trend [32,33,54,55,56,57,58,59]. Geographic patterns in temperature requirements for germination and germination rate are equally diverse [36,51,53,60,61,62,63,64,65].

Such within-species phenotypic variation has frequently been interpreted as reflecting genetic variation that has arisen through natural selection in response to variation in local environmental pressures along latitudinal/altitudinal/longitudinal gradients. However, there is limited evidence that phenotypic variation along these gradients can be explained by genetic differences [60,66,67,68]. Contrasting explanations arise, in part, because multiple seed tissues can contribute to the phenotypic variation. These tissues have different origins, serve different functions, and can be differentially influenced by the environment where seeds are produced.

**Figure 1:**
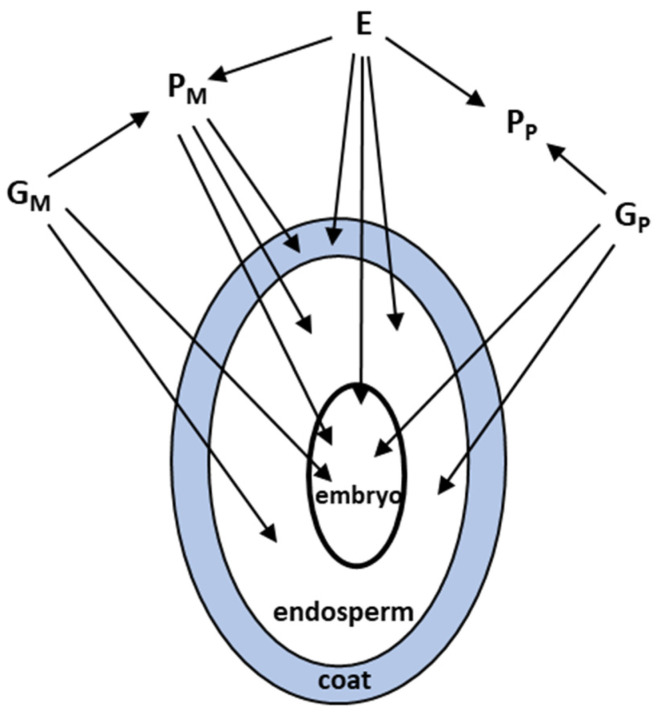
Parental contributions to the phenotype of each seed tissue: Gm = maternal direct genetic, Gp = paternal direct genetic, E = direct environmental, Pm = maternal phenotypic, and Pp = paternal phenotypic.

Three tissues typically constitute a seed, and all three have the potential to independently influence seed phenotype, e.g., mass (size, weight), % germination, germination timing, and dormancy (Figure 1, [7,9,69]). The seed coat is completely derived from maternal tissue, the embryo contains equal nuclear genetic contributions from the mother and father, and the triploid endosperm contains twice the nuclear genetic contribution from the mother than from the father, producing dosage effects. Resource availability influences seed provisioning, which typically occurs in the endosperm. Additionally, the cytoplasms of the endosperm and embryo (the zygotic tissues), which contain mitochondrial and plastid DNA, are usually only maternally inherited (See [70]) for some exceptions). A maternal phenotype can contribute materials, e.g., nutrients, hormones, and structural proteins to all three tissues, and her contributions are influenced by resource availability and other local environmental factors. Thus, maternal and paternal parents and the zygotic tissues can contribute separately, or interactively, in different ways to seed traits, and contributions could be genetic or environmentally induced. In addition, tissues could differ in their phenotypic plasticity, i.e., in their responses to local environmental change [71,72,73].

The above contributions have evolutionary and conservation implications because only the genetic variation in a trait is available for natural or artificial selection. Currently, aside from *Arabidopsis thaliana* [60,68,74], there is little supporting evidence for the hypothesis that the observed phenotypic variation in seeds across geographic ranges is explained by genetic variation. A major reason for this negligible support is that very few large-scale studies have tested the possibility that patterns could be explained by transgenerational (e.g., parental) environmental effects. For example, multiple controlled experiments have demonstrated that temperature and precipitation (primary determinants of climate) during the parental generation can affect seed phenotypes [10,74,75,76,77,78,79,80]. Because most data from large-scale geographic studies have come from tests of seeds collected directly from wild populations growing under different environmental conditions, it is not possible to rule out the hypothesis that parental environmental effects, rather than genetics, explain the geographic patterns described above. For this reason, Donohue et al. (2010) [60] pointed out that researchers need to grow the seeds gathered from wild-grown plants under uniform conditions, and then researchers should measure the properties of seeds produced by this second generation. Very few large-scale geographic studies have included this additional step [3,31,44,47,67,81,82].

Additionally, just as transgenerational environmental effects can limit one’s ability to interpret large-scale phenotypic data, so also can the absence of knowledge about the sources of genetic variation in seeds, e.g., parental vs. zygotic control. Multiple controlled experiments have detected strong maternal genetic effects on seed traits, independent of zygotic genes transmitted directly from mother to offspring and independent of maternal environmental effects [7,14,82,83,84,85,86,87,88,89]. In addition, theoretical models have demonstrated that transgenerational genetic effects can substantially impact the rate and direction of evolutionary change in a population in response to directional selection [90,91,92]. Theoretical and empirical experiments argue strongly for examining the role(s) of transgenerational genetic effects in explaining geographic patterns of seed traits.

We had the opportunity to test the presence of these effects on seed weight and germination timing in *Plantago lanceolata* L. (English, or ribwort, plantain), Plantaginaceae across its native range in Europe. *P. lanceolata* genetically varies in thermal plasticity of floral color, reflectance, and in flowering time along its latitudinal/altitudinal ranges in Europe [93]. Using seeds from a QTL experiment to explore the genetics of these thermal plasticities [94], we tested the hypothesis that northern and southern populations differ genetically in seed mass and germination timing. Specifically, we asked: (1) Are seed mass and germination timing influenced by transgenerational genetic effects? (2) Are these transgenerational effects associated with latitude? If so, what is the pattern? (3) Are the transgenerational genetic effects transmitted via the mother, father, or both? (4) Are the effects on total seed mass explained by differences in embryo/endosperm mass, seed coat mass, or both? (5) Do the effects on seed mass contribute to differences in germination timing? (6) Are the effects likely adaptive?

## 2. Results

**Figure 2:**
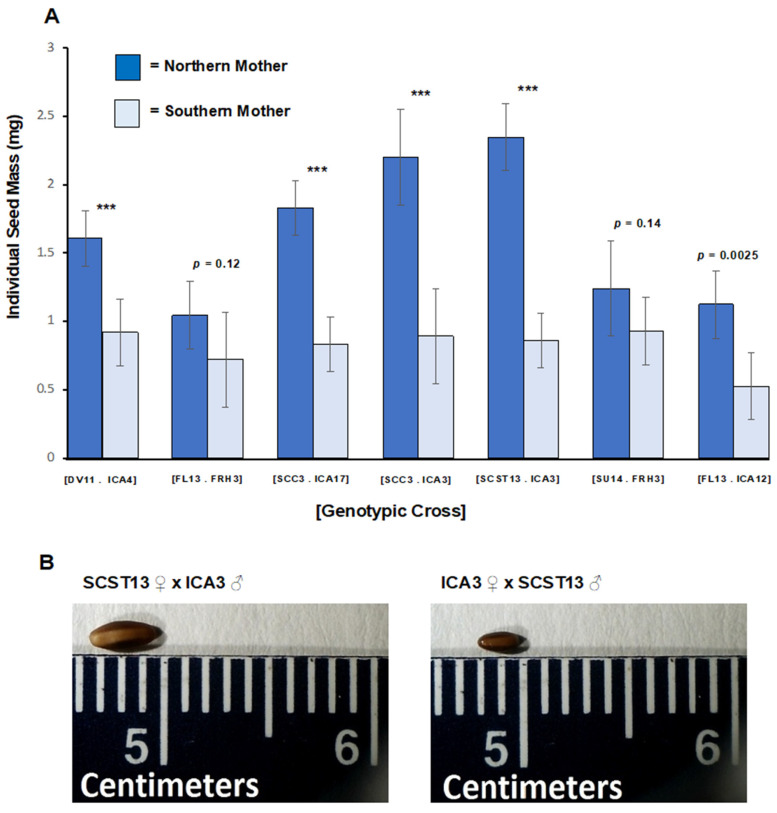
Experiment 1: (**A**) Mean individual seed mass ± 95% CI shown by reciprocal cross and maternal parent for each of seven reciprocal genotypic crosses. Genotypic crosses are identified by brackets. Each parental genotype is identified by grandparental wild population and maternal ID. Northern populations: Veno, Denmark (DV). Lund, Finland (FL), Cupar, Scotland (SCC), St. Andrews, Scotland (SCST), and Upsala, Sweden (SU); Southern populations: Hameau de St. Felix, France (FRH), Castel Volturno, and Italy (ICA). Dark blue = northern mother/southern father; Light blue = southern mother/northern father. *** = *p* < 0.0001. (**B**) Representative seeds produced by a northern mother (SCST 13) and by a southern mother (ICA 3) for a genotypic cross.

In Experiment 1, we found strong evidence of a transgenerational genetic effect on seed mass. Reciprocal crosses significantly differed from each other (F = 22.31, *p* < 0.0001). Northern mothers produced heavier and larger seeds than did southern mothers, and maternal latitude explained 57% of the variance in seed mass (Figure 2 mean seed mass + s.e. of northern mothers: 1.63 ± 0.13 mg; southern mothers: 0.82 ± 0.04 mg). The difference between seeds produced by northern vs. southern mothers within a genotypic cross was highly statistically significant for five of seven crosses. Differences in seed mass among northern mothers were statistically significant (F = 12.39, *p* = 0.002), whereas differences among southern mothers were not (F = 2.67, *p* = 0.11). Mass was positively correlated with maternal latitude (r^2^ = 0.67, *p* = 0.024).

**Figure 3:**
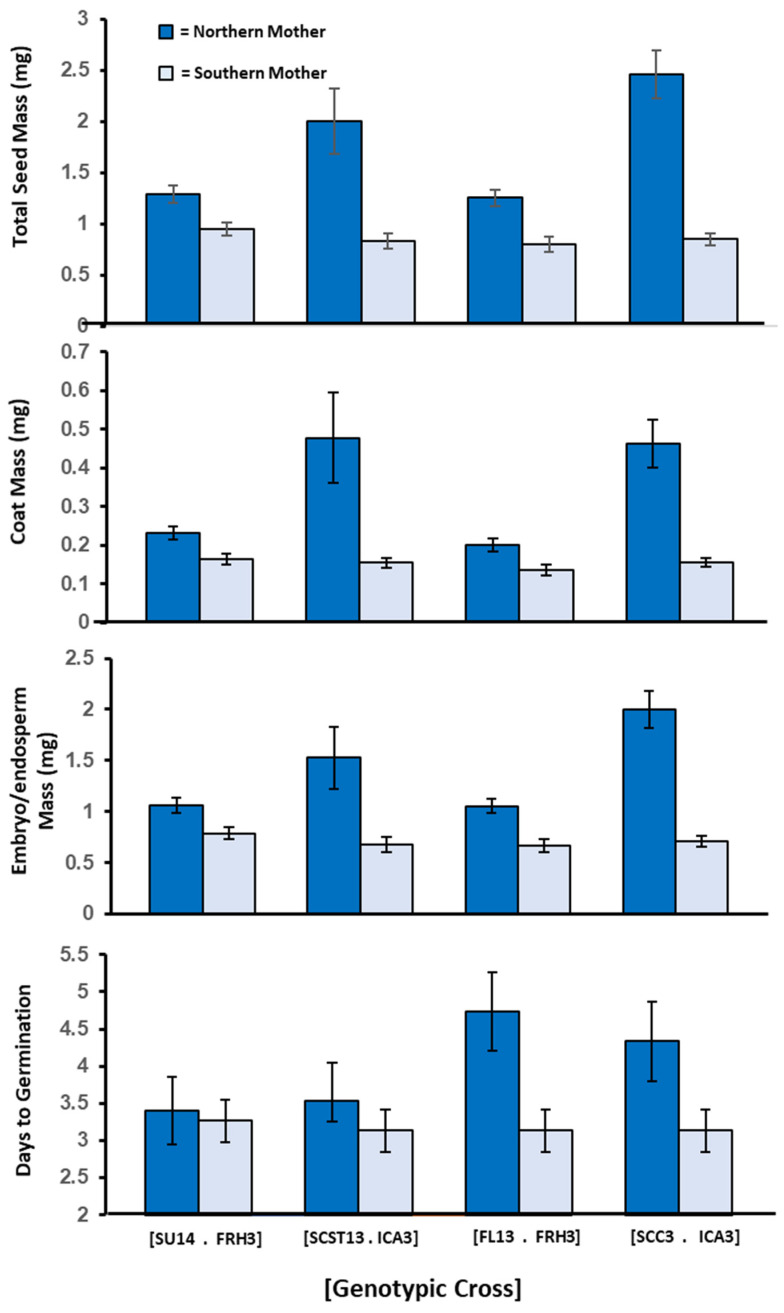
Experiment 2: Mean ± 95% CI for seed components and days to germination shown by reciprocal cross and maternal parent for each of four reciprocal genotypic crosses. Genotypic crosses are identified by brackets. Each parental genotype is identified by grandparental wild population and maternal ID. Dark blue = northern mother/southern father, Light blue = southern mother/northern father. Statistical difference between mothers within genotypic crosses were not determined, as in Figure 3, because we lacked information about clonal cross for all reciprocal pairs in this experiment.

In Experiment 2, parental latitude explained 60% of the variance in whole seed mass, 52% in coat mass, and 61% in embryo/endosperm mass (Figure 3). Northern mothers produced statistically heavier seed coats (F = 6.64, *p* = 0.042) and embryo/endosperm mass (F = 9.52, *p* = 0.021) than did southern mothers. Both coat and embryo/endosperm contributed to the statistically significant difference in total seed mass between northern and southern mothers (F = 9.20, *p* = 0.023; mean ± S.E.: Northern = 1.75 ± 0.08 mg, Southern = 0.86 ± 0.02 mg). In addition, germination occurred later in seeds of northern mothers than in southern mothers. The difference in timing was statistically significant (F = 6.72, *p* = 0.041). Mean germination time for seeds of northern and southern mothers was 4.000 days and 3.167 days, respectively (range = 3–6 days; germination = 100% on day 7). Germination timing was significantly correlated with seed mass (Pearson correlation coefficient = 0.933, *p* = 0.002). Larger seeds germinated later. Although we lacked information to statistically test for differences within genotypic crosses, we can see that the mean differences between northern vs. southern mothers with genotypic crosses in this experiment paralleled the statistically significant differences observed in Experiment 1.

## 3. Discussion

If we are to understand the nature of large-scale phenotypic variation in seeds within species, we need to determine how seed traits vary genetically across geographic gradients, e.g., in [24,60]. We have found seven studies that have tested for genetic differences in seeds produced by plants that were derived from wild populations along a latitudinal gradient but then had been grown in a common environment [3,31,44,47,67,68,81]. Four studies provide evidence consistent with genetic variation in seed mass and % germination associated with latitudinal, longitudinal, altitudinal and/or seasonal temperature gradients, exception: [47]. Wagmann et al. (2012) [67] and Debieu et al. (2013) [68] found evidence of a latitudinal gradient of parental genetic effects on seed dormancy. Because we used reciprocal crosses for our experiments, our results add new information about the nature of such genetic variation along latitudinal gradients.

Results of our reciprocal crosses combined with data from earlier studies provide evidence that latitudinal variation in seed size and germination timing can be directly influenced by parental genotype. In quantitative genetic terms, latitudinal differences between northern and southern genotypes explained the total variance in seed mass and germination timing in *P. lanceolata* more strongly than did the differences between the parental nuclear contributions to seeds. We cannot eliminate the possibility of epigenetic effects, which are poorly understood [74,95]. However, our data are derived from populations experimentally grown in similar environments. In addition, an earlier experiment with *P. lanceolata* demonstrated that statistically significant transgenerational temperature effects on seed mass disappeared in the grandoffspring generation [79]. Groot et al. (2017) [74] observed similar thermal effects using multiple accession of *Arabidopsis thaliana* from different portions of its species range. Taken together, our results for *P. lanceolata* provide additional support that transgenerational genetic effects should be considered as an explanation for variation in seed traits in other species across their geographic ranges. To test for transgenerational genetic effects, we encourage future studies to incorporate reciprocal crosses of genotypes from different regions into experimental designs. The reciprocal cross design has been used to detect the parental source of transgenerational genetic effects in crop species (e.g., see references in [96]). This type of cross would allow researchers to test for parental genetic effects along large-scale geographic gradients, and to test for transgenerational epigenetic effects, if crosses were conducted in multiple controlled environments.

Seed mass has most often been found to be determined by the maternal parent [97], and recent molecular studies support this. For example, maternal interactions between signaling pathway and phytohormones can function to regulate seed size in all three seed tissues [98,99,100,101]. Our results provide support for maternal genetic control. Parental latitude significantly affected the seed coat, which is solely maternal tissue. Because the thickness of the seed coat can influence dormancy and timing of germination [9,71,78], the maternal genetic effect on the seed coat may also indirectly affect germination timing. The observed effect on the embryo/endosperm tissue suggests either an endosperm dosage effect or a cytoplasmic effect mediated by the mother. However, significant sire contributions to germination timing have been detected in other species [89,102,103], and therefore, cannot be eliminated from consideration in our data analysis. Because of the limited number of reciprocal crosses available to us, we were not able to test for the presence of paternal genetic effects. Such sire contributions might explain why the patterns for seed mass and germination timing in Experiment 2 differ (Figure 3). Results suggest that using a more complete reciprocal design, and diallel crosses, in future large-scale geographic studies of seeds of species, generally, would be useful because they would allow researchers to partition transgenerational effects into maternal vs. paternal components.

**Figure 4:**
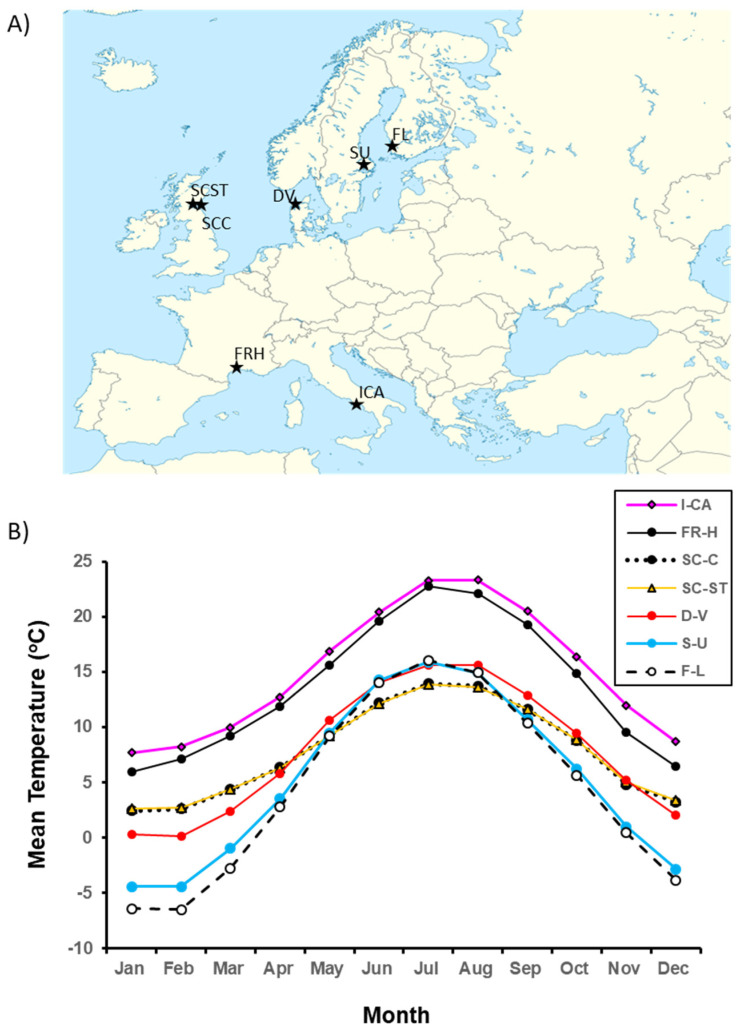
(**A**) Locations of original field populations from which the experimental genotypes were descended. Veno, Denmark (DV); Lund, Finland (FL); Cupar, Scotland (SCC); St. Andrews, Scotland (SCST); Upsala, Sweden (SU); Hameau de St. Felix, France (FRH); Castel Volturno, Italy (ICA). (**B**) Estimated 30-year mean monthly temperatures for the seven source populations (See Lacey et al., 2010 [106] for determination method).

Little is known about the evolution of transgenerational control of seed traits along latitudinal and altitudinal gradients [60,74,95,104]. Two adaptive hypotheses seem relevant to seed mass and germination timing. Maternal genetic control of seed size and germination timing is likely to be increasingly favored (1) first, as the harshness of the environment increases, i.e., with increasing latitude and altitude, and (2) second, in variable environments. Such control could offset the negative effects of a temporary extreme environmental influence, e.g., a bad year for a perennial, and thus serve as a bet-hedging mechanism against negative environmental fluctuations. Empirical results from multiple studies of *P. lanceolata*, including this one, provide support for both hypotheses. In this study, we observed that northern mothers produced larger seeds with delayed germination, in contrast to southern mothers. Maternal seed mass was positively correlated with latitude. There was also an association between maternal seed mass and the grandparental population’s thermal environment during growing season. Scottish mothers produced the largest seeds, on average, and were derived from populations having the coldest mean monthly temperatures during the growing season (compare Figure 4B with Figure 2A). Harsher conditions for seedling establishment should favor larger seeds [8,34,42,53,105]. In addition, delaying germination in the northern portion of the species range is likely to be adaptive. In the southern portion of the range in North America, seed dispersal begins in summer, with germination and seedling establishment occurring in autumn and the following spring, depending on rainfall. Further north and at higher altitudes, autumnal growing seasons are cooler and shorter. This latitudinal/altitudinal pattern characterizes the species’ range in Europe [106]. Thus, with increasing latitude/altitude, the duration of time when temperatures suitable for successful seedling establishment in autumn diminish. This diminution should selectively favor mothers that both postpone offspring germination until the following spring and provide germinating offspring with more nutrients in the endosperm to facilitate winter survival and spring seedling establishment. Such a hypothesis needs testing.

Multiple empirical experiments with *P. lanceolata* now support the hypothesis that maternal genetic effects, maternal environmental effect and maternal genetically based thermal plasticity influence offspring. Seed traits can be affected by (1) temperatures during the maternal generation, (2) maternal genotype, (3) thermal plasticity of the mother, and (4) maternal genotype by temperature interactions [75,78,84]. The positive association between the maternal control of seed traits and latitude parallels the latitudinal pattern for genetically based maternal thermal plasticity in flower color [93,94,106]. Coupling this maternal thermal plasticity with maternal genetic controls could synergistically enhance offspring establishment in cooler and temporally variable environments, and secondarily provide environmental flexibility when dispersing to a new habitat.

Finally, transgenerational controls of seed traits have important implications for facilitating species conservation and preservation. First, conservation biologists often collect seeds from multiple populations for seed preservation or breeding. Transgenerational effects can influence the success of collection methods. In the extreme case of purely parental genetic determination, natural selection on seeds occurs completely via selection on parental genotypes, not on the zygotic components of a seed [85,107]. Therefore, if seed traits are strongly determined by transgenerational effects in outcrossing species, one should sample many mothers in each population. If traits are determined by the zygotic genes, sampling multiple mothers becomes less critical because within a maternal family, genetic diversity would be greater. In addition, transgenerational control might change across geographic gradients.

Second, the strength of transgenerational control of seed traits could impact the success of breeding cultivars for habitat and species restoration. Breeding is increasingly being used in the restoration of degraded habitat and for transplantation beyond a current species’ range [9,23]. Artificial selection in the absence of knowledge about parental vs. zygotic control of seed traits could produce unintended results. For example, intra-family selection can be used effectively to preserve genetic diversity if seed traits are controlled by zygotic nuclear genes. However, if traits are controlled uniparentally via parental genetic effects, then one would need to use inter-family selection to maintain genetic diversity. In addition, one should consider the possibility that parental genetic effects can retard responses to selection. These effects can theoretically produce time lags in the responses to selection and/or accentuate or reduce responses [90,91,108]. In the absence of information about the causes of seed trait variation, generations of breeding in a common environment are likely to reduce genetic diversity [24,109].

## 4. Materials and Methods

### 4.1. Biology of Plantago lanceolata

*Plantago lanceolata* L. (English, or ribwort, plantain), Plantaginaceae, is a temperate, weedy, herbaceous perennial rosette species, native to Eurasia but now well established in disturbed areas, lawns, and grasslands in North America [93] and other continents. Photoperiodically controlled flowering occurs under long days. Flowers are protogynous, obligately outcrossing [94,110] and predominately wind-pollinated [93]. Thermal plasticity in flower color is positively correlated with latitude and altitude. These correlations are best explained by local adaptation in response to the duration of the reproductive season and to the amount of time during the reproductive season when temperatures are low [91,111,112]. Seeds, produced over several months, do not naturally disperse far from the mother.

Many studies have documented genetic variation in seed traits in and among local populations [23,77,106,113,114,115,116,117,118,119,120]. Parental environment and maternal genotype in *P. lanceolata* can influence seed mass and germination, as evidenced by both controlled and field experiments [73,77,82,114]. Studies have provided evidence that (1) cool parental temperature increases total seed weight and reduces germination relative to the effects of warm parental temperature [73,82], (2) maternal families differ in thermal response [73,82], and (3) an increase in seed coat mass, but not embryo/endosperm mass, explains the increase in total seed mass at cooler temperature [76]. These results help to explain the observation by Mondoni et al. (2011) [52] that seeds collected from an alpine population were phenotypically heavier than those from a lowland population.

### 4.2. Experimental Design

Our two experiments derive from a QTL experiment, in which Marshall et al. (2020) [94] explored the genetic architecture of differences in thermal plasticity in flower color and flowering time between northern and southern European populations. Because we used F1 seeds from this experiment, we summarize aspects of the QTL experiment and refer readers to Marshall et al. (2020) [94] and to an earlier experiment describing the latitudinal variation in thermal plasticity in flower color (Lacey et al., 2010 [106], for more details). The northern and southern parents used in the QTL study displayed high and low thermal plasticity, respectively, and represented plasticity extremes found in a sample of 29 European P. lanceolata populations. Parents in the QTL study were derived from wild European populations differing in thermal regime and duration of the growing season (Figure 4, see also Lacey et al., 2010 [106]). To reduce maternal environmental effects, Lacey et al. (2010) [106] had induced genotypes to flower and set seed while keeping the wild populations separated but in similar controlled environments. To maintain genetic variation within populations, multiple plants were grown per population. Populations grew vegetatively in a greenhouse and then isolated in growth chambers and separate greenhouse rooms set at 22 °C, 16-h day/17 °C, 8-h night to allow for random within-population wind pollination and seed production. Seeds produced from these outcrossed plants were collected by the maternal family and germinated; the resulting 29 populations were maintained in the greenhouse for subsequent experiments. In 2012, Marshall et al. (2020) [94] reciprocally crossed combinations of four genotypes from 2 northern and 2 southern populations (4 genotypes total). These parental genotypes produced the F1 seeds used in our experiments (Figure 4). Multiple clones of parental genotypes were used to produce F1 seeds. For all crosses, the single growth chamber was set at 20 °C, 16-h day/15 °C, 8-h night. F1 seeds were harvested, counted, and stored at room temperature in the lab for several months, which allowed time for after-ripening (2–3 months, Lacey, pers. observation) to be completed. Then we sampled seeds for the two experiments, described below. For both, we chose seeds that were brown and shiny and avoided seeds that were black and flat, which indicated that the seeds had been aborted.

Experiment 1: Five northern parental genotypes (one per population) were reciprocally crossed with five southern genotypes (one from a French population and four from an Italian population). The four genotypes from the Italian population were descended from different grandmothers. The two southern populations showed strong genetic differences between them [93]. For each reciprocal cross, we randomly sampled 20 seeds per clonal cross (1–3 clonal cross per reciprocal) and weighed seeds, each to the nearest 0.001 mg.

Experiment 2: After completing Experiment 1, there were enough seeds from 4 genotypic crosses to conduct a second experiment. Using these crosses again, we collected data on total seed mass, coat mass, endosperm/embryo mass, and days to germination. Fifteen seeds per reciprocal cross were selected randomly and independently of clone. After weighing each seed individually per reciprocal cross, we placed 3 seeds in each of 5 petri dishes lined with filter paper that was saturated with water. The petri dishes were placed in a growth chamber set at 20 °C, 16-h day/15 °C, 8-h night, and water was added as needed to keep the filter paper moist. Seeds were checked daily. We recorded the day of appearance of a radicle protruding from the seed coat. As germination continued, we collected the seed coats, which drop off after germination. Coats were dried and stored at room temperature for a week after all germination had ceased. All seeds germinated within 7 days from the day of placement in the growth chamber.

### 4.3. Statistical Analysis

For both experiments, we used general linear models (SAS, version 9.4) to look for the presence of transgenerational genetic effects. Given that we were working with a specific group of parental genotypes, we treated the independent factors as fixed. For experiment 1, data were gathered from 7 pairs of northern/southern genotypic crosses (14 reciprocal crosses), each defined by a maternal and paternal genotype, and 1–3 clonal crosses per reciprocal. The average seed mass per clonal cross served as the replicate values for reciprocals. We assessed the seed mass differences for: (1) 14 reciprocal crosses, (2) reciprocals having southern mothers, (3) reciprocals having northern mothers, and (4) reciprocals within each genotypic cross. Analyses based on paternal latitude produced the same results as those for maternal latitude. We present our results based on maternal latitude because many controlled experiments have demonstrated that seed traits are more often determined by the mother than the father (discussed above). Southern European mothers were represented by the French and Italian genotypes, while northern mothers were represented by the Danish, Scottish, Finnish, and Swedish genotypes. We also regressed seed mass on maternal latitude. Latitude values were extracted from Lacey et al. (2010) [106].

For Experiment 2, we tested the hypothesis that seed mass, coat mass, embryo/endosperm mass, and days to germination were associated with maternal latitude. The coat mass was subtracted from seed mass to determine embryo/endosperm mass. Because information about parental clones within reciprocal crosses was missing, we treated the data conservatively and used the mean values of reciprocal crosses as independent replicates for the analyses. For the same reason, we could not statistically test for differences in maternal latitude within genotypic crosses. We could, and did, determine the extent to which seed mass and days to germination were linearly related.

## 5. Conclusions

Multiple biologists have argued that we must begin to examine how populations within species vary genetically across geographic and environmental gradients [24,60,63]. This requires taking advantage of experimental designs that focus specifically on characterizing the genetic control of seed traits. This means not only taking seeds collected from wild populations through one generation in a common environment before measuring seed traits [60]. It also means conducting reciprocal crosses of plants derived from the same and different geographic regions or environmental regimes. Reciprocal and more extensive diallel crosses can inform us about the relative strengths of transgenerational genetic and environmentally induced effects, and zygotic genetic effects and their interactions on the phenotypic expression of seed traits. It is the phenotypic expression of these seed traits that determine subsequent life history traits and fitness in a given environment. Understanding these relative strengths will improve our ability to predict how populations of species are responding and will continue to respond in the short term and evolutionarily to global environmental change.

## Data Availability

Data will be deposited in Dryad.
